# IL-17 in osteoarthritis: A narrative review

**DOI:** 10.1515/biol-2022-0747

**Published:** 2023-10-14

**Authors:** Juan Xiao, Ping Zhang, Fang-Lan Cai, Cheng-Gen Luo, Tao Pu, Xiao-Li Pan, Mei Tian

**Affiliations:** Department of Rheumatology and Immunology Department, Affiliated Hospital of Zunyi Medical University, Zunyi 563000, China; Department of Rheumatology and Immunology Department, Zunyi Medical University, Zunyi 563000, China; The First School of Clinical Medicine, Zunyi Medical University, Zunyi 563000, China; Department of Nephrology and Rheumatology, Moutai Hospital, Renhuai 564500 Guizhou, China

**Keywords:** osteoarthritis, interleukin-17, joint cartilage, synovium, target

## Abstract

Osteoarthritis (OA) is a painful joint disease that is common among the middle-aged and elderly populations, with an increasing prevalence. Therapeutic options for OA are limited, and the pathogenic mechanism of OA remains unclear. The roles of cytokines and signaling pathways in the development of OA is a current research hot spot. Interleukin (IL)-17 is a pleiotropic inflammatory cytokine produced mainly by T helper 17 cells that has established roles in host defense, tissue repair, lymphoid tissue metabolism, tumor progression, and pathological processes of immune diseases, and studies in recent years have identified an important role for IL-17 in the progression of OA. This narrative review focuses on the mechanisms by which IL-17 contributes to articular cartilage degeneration and synovial inflammation in OA and discusses how IL-17 and the IL-17 signaling pathway affect the pathological process of OA. Additionally, therapeutic targets that have been proposed in recent years based on IL-17 and its pathway in OA are summarized as well as recent advances in the study of IL-17 pathway inhibitors and the potential challenges of their use for OA treatment.

## 1 Introduction

Osteoarthritis (OA) is one of the main causes of joint stiffness, pain and disability in the middle-aged and elderly populations, with hip and knee OA ranking 11th on the global list of disabling factors [1]. The pathogenesis of OA is complex, and the etiology is not completely clear. It is currently believed to be the result of a combination of risk factors, mainly including advanced age, obesity, joint misalignment, increased pressure load, genetics, and inflammation, with advancing age and obesity being the most prominent [2]. OA is characterized by progressive cartilage degeneration followed by the gradual development of synovial inflammation, subchondral bone sclerosis, bone redundancy formation, degeneration and tearing of the meniscus, inflammation and fibrosis of the infrapatellar fat pad [3,4], and even involvement of the entire joint [5,6]. Chronic joint pain severely affects the quality of life of OA patients, which can lead to the development of depressive states, increasing the probability of self-harm and suicide [7,8]. With the continued aging of the population and the increase in obesity in recent years globally, the reported number of OA cases increased from 248 million in 1990 to 528 million in 2019, following an increasing trend each year [9]. The current prevalence of OA in China is approximately 15%, and the condition affects up to 50% of people over 60 years of age, with a greater prevalence in women versus men and in rural populations vs urban populations, with geographical variation [10]. This high prevalence results in a huge consumption of medical resources and a huge burden on individuals, the economy, and society [11,12]. However, the current clinical treatments for OA are mainly aimed at relieving pain, protecting joint function, and slowing disease progression, and no non-surgical treatment strategy is available to control or reverse OA [13]. Although, patients with end-stage OA can choose joint replacement surgery, this treatment option is associated with risks of postoperative bleeding, infection, thrombosis, and persistent joint pain, and some patients are unable to tolerate the surgical procedure [14]. Therefore, research to understand the pathogenesis of OA in depth and to identify targets and drugs for effective OA treatment is needed to address urgent clinical problems at present.

OA is the result of an imbalance between cartilage synthesis and degradation that occurs under the combined effects of mechanical and biological factors, such as trauma, inflammation, aging, and various genetic, immune, and metabolic factors [7,15]. Articular cartilage includes chondrocytes and cartilage matrix (mainly containing water, type II collagen, and proteoglycans). Under normal conditions, type II collagen interlinks with other collagens in the extracellcular matrix (ECM) in the form of cross-linked microfibils to maintain the biomechanical skeleton of articular cartilage, and the ECM has a strong water retention capacity (hydrophilic and negatively charged), thus allowing frictionless movement of the articular surfaces and eliminating the crowding forces generated by pressure loads on the articular surfaces [16]. In addition, proteoglycans protect the articular surfaces from compressive deformation by regulating the fluid pressure in the cartilage tissue. With the occurrence of OA, interaction between chondrocytes and ECM is altered, proteoglycans and type II collagen are degraded, chondrocyte catabolism is enhanced, cartilage surfaces are compressed and eroded, and inflammatory factors are increased, leading to synovial inflammation and further aggravating the development of OA [17,18,19].

Chondrocyte differentiation and apoptosis as well as cartilage matrix synthesis and degradation are dynamically balanced to maintain cartilage homeostasis. Disturbance of cytokine homeostasis disrupts this intrachondral homeostasis, and thus, is one of the most important factors in the pathogenesis of OA [20]. Researchers have found elevated levels of tumor necrosis factor alpha (TNF-α), interleukin (IL)-1, and IL-6 in the synovial fluid, synovium, and cartilage ECM of OA patients, which suggests that low-level inflammation is involved in the pathogenesis of OA [21,22,23]. In addition, the infrapatellar fat pad, which is as rich in vascular and neural tissue as the synovium, appears to be an anatomical-functional unit with the synovium, and is thought to be one of the causes of OA joint pain [24,25]. The infrapatellar fat pad also produces pro-inflammatory cytokines and chemokines that induce synovial inflammation and promote OA progression [26]. These cytokines are known to stimulate chondrocytes, disturb the balance of anabolic and catabolic metabolism, induce high expression of matrix metalloproteinase 3 (MMP3), MMP13 and other factors, and contribute to cartilage matrix degradation [27,28]. They also stimulate synovial cells to release proteases, causing synovial inflammation and promoting bone resorption [28], which induces progressive degradation and destruction of articular cartilage. Accordingly, the important roles of cytokines in the pathogenesis of OA have been confirmed.

In recent years, the relationship between the pleiotropic inflammatory cytokine IL-17A (also commonly referred to as IL-17) and OA has attracted considerable attention. Previous studies showed that IL-17 is associated with host defense, tissue repair, lymphoid tissue metabolism, and tumor progression [29], and IL-17 has been most extensively studied in relation to autoimmune diseases, such as rheumatoid arthritis (RA) [30], ankylosing spondylitis [31], and psoriatic arthritis [32]. The circulating IL-17 level was shown to play an important role in the pathogenesis and progression of inflammatory arthritis [33]. Synergy between IL-17 and TNF-α has been demonstrated to activate the production of proinflammatory mediators, such as IL-1β, IL-6, IL-8, prostaglandin E2 (PGE2), and MMPs, to promote the progression of early inflammation to chronic arthritis [34]. In the context of OA, IL-17 affects the inflammatory response, complement production, hypoxia response, angiogenesis, and glycolytic pathways in chondrocytes and synovial fibroblasts [35]. IL-17 may play a crucial role in the pathogenesis of OA and is closely associated with joint pain in OA patients [36]. Significant correlation between IL-17 expression and cartilage defects and bone marrow lesions was observed in the serum of patients with knee osteoarthritis (KOA), and a positive correlation was also observed between IL-17 expression and the severity of KOA [37,38]. Several animal models have also confirmed the role of IL-17 in inflammatory arthritis [39,40,41], and injection of IL-17 into the rabbit knee joint induces a model of OA similar to that induced by the Hulth method [42]. Therefore, This review article summarizes current knowledge regarding the mechanism of action of IL-17 in OA and discusses the potential challenges of using IL-17 inhibitors for the treatment of OA.

## 2 Introduction of IL-17

**Figure 1:**
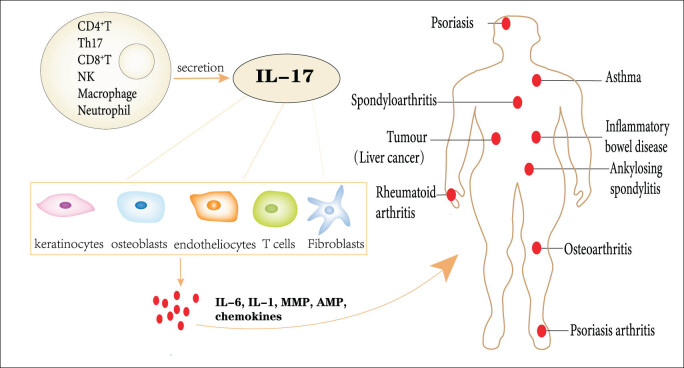
IL-17 is produced by Th17 cells, CD8^+^ T cells, γδ T cells, NKT cells, etc. IL-17 acts on keratin-forming cells, fibroblasts, osteoblasts, endothelial cells, and immune cells and participates in the pathological processes of tumor development, immune diseases, and inflammation by inducing production of inflammatory factors, inflammatory mediators, and MMPs.

The IL-17 family is a class of structurally similar inflammatory molecules that includes six cytokines, IL-17A, IL-17B, IL-17C, IL-17D, IL-17E (also known as IL-25), and IL-17F. They are mainly derived from T helper 17 (Th17) cells, CD8^+^ T cells, γδT cells, and natural killer (NK) T cells [43] and can also be produced by neutrophils and macrophages during inflammation (Figure 1) [44,45]. The most well-known one is IL-17A (referred to hereafter as IL-17 unless otherwise stated). To achieve their biological effects, members of the IL-17 family must bind to the corresponding receptor (R) complex on the cell surface to regulate gene transcription. The IL-17 recepter (IL-17R) family has five receptor subunit members, which are labeled IL-17RA, IL-17RB, IL-17RC, IL-17RD, IL-17RE, of which IL-17A and IL-17F exist as homologs or heterodimers and act together in a complex formed with IL-17RA and IL-17RC. Interestingly, IL-17RA exerts pleiotropic functions by binding and interacting with IL-17RB, IL-17RC, IL-17RD, and IL-17RE [46,47]. In the inflammatory environment, IL-17 can be active against various cells, including keratin-forming cells, fibroblasts, osteoblasts, endothelial cells, and immune cells [48], and is involved in the pathological processes of inflammation, autoimmunity, tumorogenesis, and metabolic disorder through the production of various molecules, such as inflammatory factors (IL-6, IL-1, TNF), chemokines, antimicrobial peptides (AMPs), MMPs, and acute phase proteins (Figure 1) [49].

## 3 IL-17 signaling pathway

**Figure 2:**
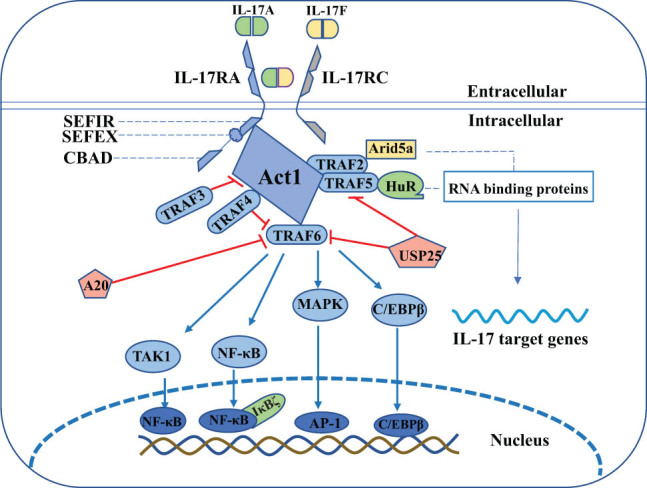
IL-17 signal transduction pathway.

IL-17RA consists of an extra-membrane fibronectin-like structural domain, an intracytoplasmic SEF/IL-17(SEFIR) structural domain and a distal activation structural domain (CBAD). In addition, IL-17RA also has SEFEX, an extension sequence of SEFIR [50]. The signal transduction and negative regulatory pathway of IL-17 consists of four main steps: (1) IL-17 (IL-17A, IL-17A/F, IL-17F) binds to receptor complexes formed by IL-17RA and IL-17RC and induces binding of the receptor SEFIR structural domain to the multifunctional signaling protein Act1 (with E3 ubiquitin ligase activity). Act1 is critical in IL-17 signaling pathway-dependent autoimmune and inflammatory diseases, and IL-17-induced expression of inflammation-related genes is suppressed when Act1 is defective [51]. (2) Act1 rapidly recruits and ubiquitinates TNF receptor-associated factor 6 (TRAF6), a critical step in signaling pathway transduction. TRAF3 and TRAF4 can negatively regulate this pathway by interfering with the coupling between Act1 and TRAF6. Additionally, deubiquitinating enzymes A20 and USP25 can remove the ubiquitination disability of TRAF6 to similarly prevent TRAF6 from binding to Act1. Meanwhile, USP25 also inhibits TRAF5 activity and affects post-transcriptional RNA stability. (3) TRAF6 promotes the activation of mitogen-activated protein kinase (MAPK)/AP-1, C/EBPβ, and δ transcription factor, and through transforming growth factor beta (TGF-β)-activated kinase 1 (TAK1), phosphorylates nuclear factor kappa B (NF-κB), targeting an important transcriptional target NF-κB inhibitor zeta (IBζ), which is involved in psoriasis development. (4) Act1 recruits TRAF2/5 and binds HuR and Arid5a to promote post-transcriptional RNA stabilization (Figure 2) [52,53]. In conclusion, IL-17 signaling is very complex, and the above description summarizes only the main processes. Many specific molecular mechanisms and branches remain to be further explored.

## 4 Role of IL-17 in the pathogenesis of OA

**Figure 3:**
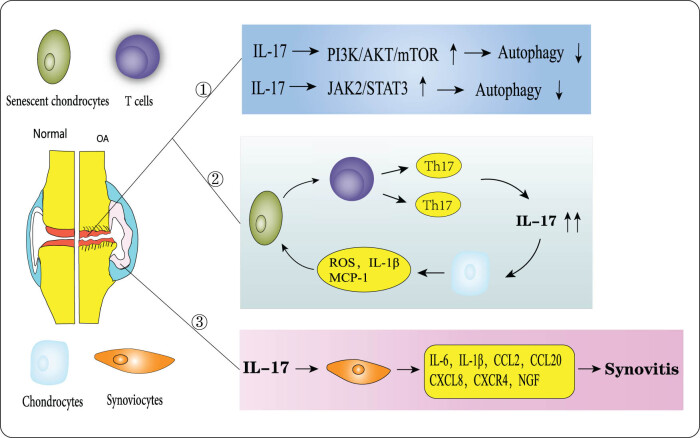
Mechanism of action of IL-17 within articular cartilage and synovium in OA. (1) IL-17 reduces autophagy through activation of the PI3K/AKT/mTOR and JAK/STAT3 signaling pathways; (2) IL-17 promotes chondrocyte senescence in OA, and senescent chondrocytes induce differentiation of naive T cells to Th17 cells in a vicious cycle; and (3) IL-17 promotes synovial inflammation.

The etiology of osteoarthritis remains incompletely understood, but various molecular mechanisms have been shown to be involved in this pathological process, including the JAK/STAT signaling pathway [54], PI3K/AKT/mTOR signaling pathway [55,56], p38 MAPK/c-Fos/AP-1 pathway [57], and Wnt/β-linked protein pathway [58]. These signaling pathways have been found to mainly affect the OA pathological process by regulating chondrocyte survival, subchondral bone remodeling, and synovial inflammation. Interestingly, IL-17 is also involved in the degeneration and destruction of articular cartilage and synovial inflammatory processes in the pathology of OA (Figure 3).

### 4.1 Cartilage

The degeneration and destruction of articular cartilage are recognized as central to the progression of OA [59] along with the involvement of IL-17 and its pathways, mainly in the form of effects on chondrocyte autophagy, senescence, and cartilage matrix [41].

Autophagy is a stress response mechanism for cell survival that involves removal of intracellular microorganisms through lysosomes and degradation of dysfunctional or damaged organelles and proteins, and is an important system for energy and nutrient metabolic homeostasis within the body [60,61]. Abnormal cellular autophagy can advance the progression of chondrocyte senescence and apoptosis, with peroxide production and mitochondrial dysfunction being the main mechanisms [62]. Previous studies by our group and others have confirmed that autophagy, as a form of cell death, is closely associated with OA progression [63,64].

Furthermore, autophagy and inflammation are two biological processes important for cells in physiological and pathological states, with autophagy regulating innate and adaptive immune responses and, conversely, immune response-induced cytokine release regulating autophagy levels [65,66,67]. IL-17 is known to influence autophagy in two ways. (1) IL-17 promotes autophagy: IL-17 can induce autophagy in B cells in vitro by increasing Erk1/2 phosphorylation, as evidenced by an increase in the activity of autophagy proteins Beclin-1 and P62 on the ubiquitin proteasome system, and by an increase in the anti-apoptotic capacity of B cells [68]. In contrast, 3-MA, an inhibitor of autophagy, significantly reversed the above effects of IL-17. It was found that IL-17 increased the accumulation of RAW264.7 cellular autophagy protein LC3B-II–induced autophagic activity, significantly increased the number and size of cellular autophagosomes, and promoted the antimicrobial activity of primitive macrophages [69]. (2) IL-17 inhibits autophagy expression: In normal skin fibroblasts, IL-17 stimulation significantly increased the expression of p-STAT3 and hypoxia-inducible factor-1α (HIF-1α) and increased P62 activity, suggesting an accumulation of autophagosomes (P62), leading to autophagy defects. hIF-1α inhibitor reversed the IL-17-induced autophagy downregulation [70]. The IL-17/STAT3/HIF-1α/P62 signaling axis cascade is associated with autophagy inhibition.

However, how IL-17 regulates autophagy in OA remains less well characterized. An study observed significantly elevated IL-17 expression in an IL-1β-induced OA chondrocyte inflammation model, which exacerbated autophagy defects and promoted cartilage degeneration by mediating PI3K/AKT/mTOR autophagy-related pathways [41]. In addition, stimulation of mouse osteoblasts with IL-17 was found to suppress the level of autophagy by activating the JAK2/STAT3 signaling pathway and downregulating Beclin 1, LC3, and Atg7 expression [71]. In summary, in OA, IL-17 promotes OA progression mainly by inhibiting autophagy expression, which is closely related to the PI3K/AKT/mTOR and JAK2/STAT3 signaling pathways, and targeting these two pathways and blocking the action of IL-17 may be the direction of OA treatment. Unfortunately, additional research in this specific area is lacking, and it remains unclear whether IL-17 is an independent upstream target of autophagy.

Cellular senescence also is involved in OA pathogenesis [15,72]. Chondrocyte senescence affects articular cartilage biomechanics, biochemistry, and cellular function, making articular cartilage more susceptible to damage [73]. In addition, one of the hallmarks of aging is mitochondrial dysfunction, which in OA leads to an imbalance in cellular energy metabolism and an increase in reactive oxygen species (ROS) production, exacerbating the progression of OA as evidenced by an increase in oxidative stress in articular cartilage, cytokine production, an increase in inflammation-mediated catabolism of cartilage matrix, and increases in calcification of the cartilage matrix and apoptosis of chondrocytes [74,75]. Experiments in an animal model of post-traumatic OA showed anterior cruciate ligament transection (ACLT) induced a Th17-type immune response with increased IL-17 expression and that senescent OA chondrocytes drove the differentiation of naive T cells to Th17 cells [39]. Furthermore, intra-articular injection of neutralizing antibodies to IL-17 into the OA animal model decreased the expression of the senescence marker P16 (Cdkn1α) [39]. Wang et al. reported that stimulation of chondrocytes with IL-17 induces production of reactive oxygen species, monocyte chemoattractant protein-1 (MCP-1), and IL-1β along with increased senescence-associated β-galactosidase activity, a prolonged stationary/gap phase (G0/G1) in the cell cycle, a shortened S-phase in DNA synthesis, and ultimately premature senescence of chondrocytes [76]. In summary, the Th17/IL-17 axis can accelerate chondrocyte senescence, and a reciprocal promoting relationship exists between IL-17 secretion and cellular senescence-associated protein expression. Moreover, this combined effect may induce more rapid destruction of articular cartilage than each single factor.

Chondrocytes are responsible for the synthesis and secretion of cartilage needed to form the cartilage matrix, and this activity is essential for the maintenance of cartilage homeostasis and is regulated by physical and chemical signaling within the joint that influences the physiological function of chondrocytes [77,78]. IL-17 was found to be involved in cartilage matrix synthesis and catabolic pathways [79]. Elevated IL-1β and IL-6 expression was observed in a sodium iodoacetate-induced IL-1 receptor antagonist (IL-1Ra) knockout mouse model of OA, and cartilage tissue thinning and chondrocyte reduction also were observed [80]. However, silencing of IL-17 significantly inhibited inflammatory mediator release and cartilage damage. The same study also stimulated human chondrocytes with IL-17 and found that MMP1, MMP3, and MMP13 were upregulated while SOX9 (a protein associated with chondrocyte anabolism) was downregulated, which led to increased cartilage matrix degradation and exacerbated cartilage tissue damage. Hu et al. reported that treatment of chondrocytes with recombinant IL-17 results in activation of the NF-κB and MAPK signaling pathways, upregulation of the cartilage catabolic factors IL-6, MMP3, and zinc metalloproteinase 4 (ADAMTS-4) expression, promotion of cartilage matrix degradation, disruption of homeostasis within cartilage, and aggravation of OA progression [81]. In summary, stimulation by IL-17 disrupts the balance in cartilage matrix metabolism, and the resulting feedback affects the physiological function of chondrocytes, leading to cartilage tissue degeneration. Thus, the role of IL-17 in OA is not limited to inflammatory effects, as this factor also plays a key role in the pathological progression of OA by influencing chondrocyte senescence, apoptosis, and cartilage matrix degradation.

### 4.2 Synovium

The synovium is a connective tissue located in the inner layer of the joint capsule that surrounds tendons and forms the lining of bursae and fat pads, which secrete and regulate the formation of synovial fluid, providing nutrients to the chondrocytes [82]. Injury-related molecules, products of mitochondrial dysfunction, cytokines, and metabolites in joints activate synovial cells to promote the release of large amounts of pro-inflammatory cytokines and inflammatory mediators, inducing OA-related cartilage matrix degradation and osteophyte formation [83,84,85]. Pro-inflammatory cytokines and chemokines are central regulators of synovial inflammation in OA [20,21]. Interestingly, IL-17 amplifies the synovial inflammatory effects in OA by promoting the expression of pro-inflammatory factors (e.g., IL-6 and IL-1β) and chemokines (e.g., CXCL8, CCL20, CXCL3, and CXCR4) [86]. Deligne et al. found that IL-17 and IL-22 are highly expressed in conditioned medium collected from synovial tissues of OA patients and responsible for inducing the combined release of IL-6, IL-23 and TGF-β1; upregulating MMP9 expression; and ultimately driving synovial inflammation and cartilage matrix degradation [87]. Moreover, in a clinical trial of end-stage hip and knee OA, patients with detectable IL-17 levels in synovial fluid had significantly increased levels of adipokines (leptin, resistin), IL-6, C-C motif chemokine ligand 2 (CCL2), CCL17, and nerve growth factor (NGF) [88]. These findings supported those of previous studies regarding the involvement of adipokines in cartilage degeneration, synovitis, subpatellar fat pad changes, and bone formation [89,90]. Again, IL-17 was shown to promote OA progression by increasing adipokine and inflammatory factor production. In conclusion, IL-17 itself has limited pro-inflammatory effects and cannot directly act on chondrocytes, but it exerts intense inflammatory effects by enhancing and synergizing the pro-inflammatory effects of other cytokines and inflammatory mediators, leading to cartilage degradation, matrix degradation, and synovial inflammation in OA.

## 5 IL-17–based therapeutic targets and drugs for OA

### 5.1 Targets

Levels of long non-coding (lnc)RNA cancer susceptibility candidate 2 (*lncRNA CASC2*) were found to be elevated in the blood and synovial fluid of OA patients, and lncRNA *CASC2* was shown to regulate chondrocyte proliferation and apoptosis through mediation of the IL-17 signaling pathway [91]. In human OA synovial fibroblasts (OASFs), the CCN family protein connective tissue growth factor (CCN2) inhibits *miR-655* synthesis by mediating the ILK and Syk signaling pathways, whereas *miR-655* can bind to IL-17 and directly inhibits IL-17 activity; therefore, CCN2 down-regulates *miR-655* expression and indirectly promotes IL-17 synthesis, leading to increased inflammation in OA [40]. Low levels of lncRNA growth arrest-specific transcript-5 (*GAS-5*) were shown to affect IL-17–related immune and cytokine expression and to be a potential marker of OA progression [92]. The cyclic RNA *ciRS-7/micro-RNA7 (mi-RNA7)* axis is aberrantly expressed in OA and may drive OA progression through upregulation of IL-17–mediated inflammatory responses [43]. IL-17 is the target gene of *miR-136*, the expression of which is negatively correlated with *miR-136* expression, and thus, *miR-136* can be used as a potential biomarker of KOA [93]. TRAF3 significantly inhibits IL-17–induced activation of NF-κB and MAPK, as well as the production of downstream MMPs, resulting in a protective effect against OA [49]. *miR-34a*, *miR-146a*, and *miR-181a* are mediators of adipokine-induced oxidative stress and synovial inflammation in humans with OA via NF-κB pathway expresssion in synoviocytes [94]. In conclusion, most therapeutic targets for OA based on IL-17 and its signaling pathway are related to mRNA expression, and targeting of the gene transcription–translation pathway is expected to be a new strategy for OA therapy.

### 5.2 Drugs

According to the 2019 edition of the Chinese Osteoarthritis Diagnosis and Treatment Guidelines, OA is most commonly treated currently with a combination of treatments, including health education, weight management, symptomatic drug therapy (non-steroidal anti-inflammatory drugs, NSAIDs), intra-articular sodium hyaluronate injection, physical therapy, and surgery, with the aims of relieving pain and improving joint function [13,95,96,97]. The 2019 American College of Rheumatology/Arthritis Foundation guidelines on OA of the hands, hips, and knees emphasize exercise, weight loss, self-management (tai chi), use of canes and hand orthotics and braces, oral and topical NSAIDs, intra-articular glucocorticosteroid injections, acupuncture, and heat treatments, to name a few, but none of them can cure OA [97]. These guidelines are similar to the Chinese guidelines for the diagnosis and treatment of osteoarthritis. However, NSAIDs can cause gastrointestinal, hepatic, renal, and cardiovascular side effects, and thus, their use should be limited [98]. Several new therapeutic approaches have been explored in recent years, including combination injections of nerve growth factor, stem cells, and platelet-rich plasma, but the balance of efficacy and safety for these approaches remains questionable [5,99]. Accordingly, treatment of OA remains a challenge worldwide. Multiple studies in recent years have demonstrated that regulation of Th17 cells, IL-17 expression, IL-17 signaling pathway activation, and mRNA expression of inflammatory mediators may be effective strategies for treating OA, with effects of reducing cartilage tissue damage and improving joint pain, inflammation, and function (Table 1). The most important of these effects is the control of synovial inflammation in OA.

**Table 1:** Potential IL-17–related treatments for OA

Drug name	Target	Research platform	Effect	Refs.
RA10-6	IL-17/IL-6	Mice	Suppression of OA synovial inflammation	[110]
Krocina™	Th17/IL-17	Humans (clinical trial)	Suppression of OA synovial inflammation	[111]
Resveratrol	IL-17/NF-κB	Rats	Repair of OA soft tissue damage	[111]
Platelet-rich- plasma	IL-17	Rats	Improvement of joint function, pain and inflammatory	[112,113,114]
Baccharis	IL-17	Mice	Suppression of OA immune inflammatory	[115]
Pioglitazone	IL-17/NF-κB	Human myeloid cells and tissues	Inhibition of mRNA expression of inflammatory mediators	[116]
1,25-Vit D3	Th17	Dendritic cells	Inhibition of pro-inflammatory cytokine	[117]
Dexamethas-one	Th17	Dendritic cells	Inhibition of pro-inflammatory cytokine	[118]
Apremilast	IL-17	ATDC5 chondrocytes	Inhibition of inflammatory factors, cellular senescence, ROS	[76]
Salidroside	CD4+/IL-17	Rats	Regulates inflammation and immune response	[118]

## 6 Inhibitors of IL-17

Inhibitors of IL-17 or the IL-17 signaling pathway, including Secukinumab (AIN457), Ixekizumab (LY2439821), and Brodalumab (AMG827), have been approved in recent years for clinical use for the treatment of ankylosing spondylitis [100,101], moderate-to-severe plaque psoriasis and psoriatic arthritis [102,103,104], radiographic and nonradiographic spondyloarthritis [105], and Netherton’s syndrome [106] (Table 2), and have shown excellent efficacy, safety, and tolerability. Additionally, some small molecule inhibitors of the orally available IL-17 pathway continue to be studied in the clinical research phase, such as IMU-935, an inhibitor of retinoic acid-related orphan receptor γt (RORγt), which is a major regulator of Th17 cell secretion and IL-17A/F production in innate and adaptive immunity and is involved in the regulation of bacterial and fungal immune responses [107]. As a treatment for psoriasis, IMU-935 was shown to reduce the release of pro-inflammatory cytokines and inflammatory mediators by inhibiting the RORγt/Th17/IL-17 signaling pathway [108,109], but its drug efficacy and risk of adverse events have yet to be established. Although IL-17 pathway inhibitors are not currently used for the treatment of OA, research evidence that IL-17 plays an important role in the pathological progression of OA and is positively correlated with OA severity suggests that inhibitors of IL-17 and its pathway may become the next clinical research target for OA treatment strategies.

**Table 2:** Mechanism and characteristics of drugs that inhibit IL-17 or the IL-17 signaling pathway

Drug	Target	Molecular mechanism	Refs.
Secukinumab	IL-17A	Human recombinant monoclonal antibody of IgG1/κ isotype; selectively binds to IL-17A and inhibits its combination with IL-17RA; blocks trans- duction of IL-17 signaling pathway.	[119,120]
Ixekizumab	IL-17A	Human IgG4/κ monoclonal antibody; binds to IL-17A with high affinity and selectivity to neutralize its activity; inhibits release of proinflamma- tory cytokines and chemokines.	[121]
Brodalumab	IL-17RA	Human monoclonal antibody of IgG2/κ isotype; specifically blocks IL-17R and inhibits proinflammatory signaling inducted by multiple IL-17 cytokines, including IL-17A and IL-17F.	[122]
IMU-935	RORγt	Specifically inhibits the RORγt/Th17/IL-17 signaling pathway; affects Th17 cell secretion and IL-17A/F production; inhibits release of relat-ed pro-inflammatory cytokines and inflammatory mediators.	[108,109]

Although down regulation of IL-17 and inhibition of the IL-17 signaling pathway can be beneficial in OA as mentioned above, inhibitors of IL-17 and its pathway are currently inappropriate for the treatment of OA based on the mechanism of action of IL-17 in the pathological process of OA, and many potential challenges persist. (1) IL-17 cannot act directly on chondrocytes but rather affects chondrocyte survival by indirectly upregulating or downregulating the expression of related pathways and proteins. Inhibiting IL-17 expression can reduce its role in promoting chondrocyte apoptosis and senescence but cannot block the pathological changes that occur in OA itself. (2) The pro-inflammatory effect of IL-17 itself is limited; this effect is instead, mainly achieved by synergizing and amplifying the effects of other pro-inflammatory factors and inflammatory mediators to indirectly promote synovial inflammation in OA. Thus, inhibiting the activity of IL-17 and its receptors only weakens or eliminates this amplifying effect but cannot block the occurrence of synovial inflammation. (3) OA progression is supported by many molecular mechanisms that are connected by subtle links. It is not accurate to consider IL-17 activity as an independent cause of OA. Therefore, an in-depth understanding of the relationship between IL-17 and other signaling pathways in OA is critical. Much basic and clinical research is needed to address questions like whether targeting IL-17 gene expression and inhibiting IL-17 pathway activation will have different effects, and whether the combined application of IL-17 inhibitors and blockers of other pathways will have different efficacies.

## 7 Conclusions

IL-17 is involved in the maintenance of innate and adaptive immunity, and dysregulated production of IL-17 has been shown to promote OA development from multiple perspectives. On the one hand, it promotes OA progression by regulating chondrocyte autophagy and senescence and cartilage matrix degradation, while on the other hand, it induces synovial inflammation by mediating the IL-17 signaling pathway to promote the release of inflammatory cytokines and adipocytokines. These research findings provide valuable evidence for the application of inhibitors of IL-17 and its signaling pathway in OA treatment. Additionally, IL-17 expression was found to be positively correlated with the severity of OA, and thus, it may become a new immunological indicator for evaluating the efficacy of OA treatment and may be an effective target for OA therapy. However, the following questions persist regarding the role of IL-17 in OA: (1) Does IL-17 affect chondrocyte mitosis and cell cycle progression in OA? (2) Are multiple members of the IL-17 family involved in OA development? (3) What relationships exist between IL-17 and other signaling pathways, and what are their side effects? In the future, it will be important to also investigate the impact of IL-17 on other joint tissues such as the meniscus and infrapatellar fat pad, considering that OA is a whole joint disease. A clear understanding of the specific molecular mechanism of IL-17’s action in OA and a means to target this axis accurately without affecting physiological functions could facilitate effective approaches for the prevention and treatment of OA based on IL-17 and its signaling pathways.
